# Biomodulator of Diode Laser Irradiation on Odontoblast-Like Cells by Expression of Vascular Endothelial Growth Factor-A and Transforming Growth Factor-β1

**DOI:** 10.1055/s-0042-1749155

**Published:** 2022-07-11

**Authors:** Devi E. Juniarti, Sri Kunarti, Andi A. Mardiyah, Ni M. D. Purniati

**Affiliations:** 1 Department of Conservative Dentistry, Faculty of Dental Medicine, Universitas Airlangga, Surabaya, Indonesia; 2 Specialist Program of Conservative Dentistry, Faculty of Dental Medicine, Universitas Airlangga, Surabaya, Indonesia

**Keywords:** diode laser 650-nm irradiation, biomodulator, odontoblast like cells, VEGF-A, TGF-β1

## Abstract

**Objective** This study aimed to prove that the effect of diode laser 650-nm irradiation to the expression of vascular endothelial growth factor (VEGF)-A and transforming growth factor (TGF)-β1 plays important roles in dental pulp-regulating cell proliferation, differentiation, and revascularization.

**Materials and Methods** The research was performed by randomized posttest only control group design using *Rattus norvegicus* . A total of 48 samples were provided and divided into eight groups of 6 samples each with a random-sample allocation. Each group were prepared, and perforation of maxillary first molar were done. In control groups (groups 1–4), glass ionomer cement (GIC) was used to restore the teeth, while in laser groups (groups 5–8), the teeth were irradiated with diode laser 650 nm for 40 seconds before application of GIC. Half of the groups (groups 1, 2, 5, and 6) were necropsied in 7 days, and the rest (groups 3, 4, 7, and 8) were necropsied in 14 days. Immunohistochemistry (IHC) evaluation were implemented to check the expression of both VEGF-A and TGF-β1.

**Statistical Analysis** Both data of VEGF-A and TGF-β1 expression were analyzed using a one-way ANOVA ( *α* = 0.05) with SPSS statistical software.

**Results** The study showed that the diode laser 650-nm irradiation increased expression of VEGF-A and TGF-β1, and there was a significant difference between diode laser and control group on VEGF-A expression ( *p* = 0.001) and TGF- β1 ( *p* = 0.000) on days 7 and 14.

**Conclusion** Diode laser 650 nm with 40-second irradiation time shows increment from day 7 to day 14 reflecting increase in pulp healing by modulating VEGF-A and TGF-β1 expression since days 7 to 14.

## Introduction

Recent studies about diode laser for vital pulp therapy are now developing. Main goal of this therapy is to initiate formation of reparative dentin. Vital pulp therapy can be done by application of some materials, such as calcium hydroxide, mineral trioxide aggregate (MTA), and biodentine, or even by another strategy such as laser irradiation, ozone technology, silver diamine fluoride, or others. 1 2 Diode laser 810 nm has more significant hemostatic effect and antibacterial advantage compared with chemical agents (ferric sulfate, chlorhexidine, and diluted formocresol solution). 3 Another study showed that diode laser irradiation is more effective than conventional pulp capping technique. 4 Diode laser with wavelength ranging between 810 and 980 nm can be well absorbed by hemoglobin and is suitable to decontaminate cavity and pulp coagulation in exposed pulp. 5 Dentolaser 650 nm showed that irradiation of diode laser for 40 seconds in pulsed mode increased fibroblast cell proliferation and odontoblast-like cells proliferation in animal studies. 6 7

Near-red laser spectrum with wavelength ranging approximately 630 to 675 nm or low-level laser therapy (LLLT) is often used in medical fields because of its anti-inflammation effect, analgesic, and biostimulation. LLLT is also capable to promote wound healing. 5 6 7 Several advantages of laser irradiation in vital pulp therapy compared with conventional technique are decontamination effect, hemostatic effect, and biostimulation effect. 4 Previous studies concluded that red or near-red laser (600–1,200 nm) has biological effect, 8 and biostimulator effect with energy density 0.05–10 J/cm ^2^ can promote proliferation, and laser with energy density >10 J/cm ^2^ can promote antiproliferation. 9

Healing stages in reparative dentinogenesis consist of four steps which are moderate inflammation, cell progenitor recruitment, cell progenitor proliferation, and final differentiation. 10 Pulp is an important part in reparative dentinogenesis because dental pulp acts as growth factor reservoirs. Vascular endothelial growth factor (VEGF-A), transforming growth factor-1 (TGF-1), fibroblast growth factor-2 (FGF-2), bone morphogenic protein (BMP), are the growth factors that play a significant role. These growth factors act in regulation of progenitor cells' recruitment, cell proliferation, and dentine-secreting cells differentiation. 1

Growth factor and cytokine are key of molecule signaling which controlled and regulated cellular involved in growth, homeostatic, and tissue recovery in dental pulp. Growth factors are peptide molecules responsible for signaling some cellular process that was happened after injury. Growth factors serve as signal transmitter of cell function, as stimulator or inhibitor of growth and also as differentiation modulator. Growth factor will regulate gene to control proliferation, cell differentiation, or cell secretory product. 11 VEGF-A is growth factor for dental development, angiogenesis in dentin pulp complexes, and proliferation in pulp recovery and dentin bridge formation. 6 12 TGF-β1 acts in homeostatic and tissue repair. 13 14

Diode laser irradiation as alternative treatment option for vital pulp therapy has no clear standardization about laser wavelength or exposure time. Based on that issue, *in vivo* study has done to verify whether diode laser irradiation is effective for vital pulp therapy with parameter of VEGF-A and TGF-β1 expression. Aim of this study is to prove that the effect of diode laser 650-nm irradiation to the expression of VEGF-A and TGF-β1 plays important roles in dental pulp-regulating cell proliferation, differentiation, and revascularization.

## Materials and Methods

### Experimental Design and Ethics Approval

The experimental design in this study was post-test-only control group design. The animal study (in vivo) was conducted in accordance with Government Regulation of The Republic of Indonesia Number 95 of 2012 Concerning Veterinary Public Health and Animal Welfare. Sample of the study was *Rattus norvegicus* male Wistar strain aged 8 to 12 weeks with an initial body weight of 200 to 250 g. All samples were in healthy conditions with a total sample count of 48 for eight groups ( *r* = 6). All procedures performed in this study are ethical and have been approved by the Ethics Commission of the Faculty of Dentistry, Airlangga University with approval no. 244/HRECC. FODM/V/2020.

### Laser Irradiate Preparation

**Fig. 1:**
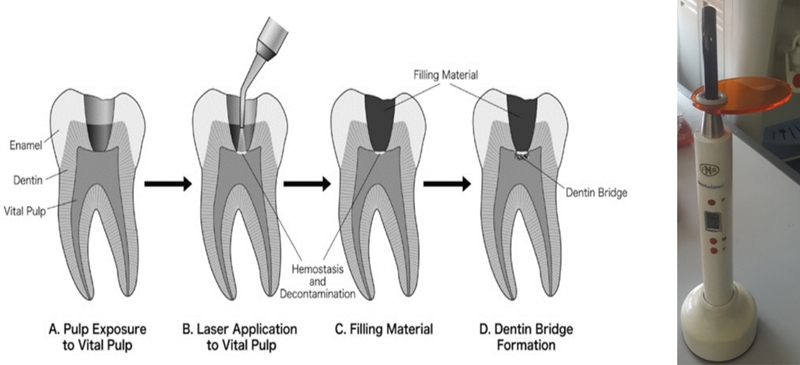
( **A–D** ) Illustration of diode laser 650-nm irradiation with 1-cm distance for 40 seconds. (adapted from Komabayashi et al). 10

The lasers used is a 650-nm diode lasers (Dentolaser 650, UNAIR, Indonesia), which has been calibrated, with a standard irradiation distance of 1 cm with a power of 22 mW, considering the area of irradiation and controlling light at the time of caliber ( Fig. 1 ). Laser is a device that irradiates light through a process of amplification which is stimulated by photon emission. Diode laser works by releasing energy in the form of photon as a result from combination of electron and electron hole in the device. This photon will be absorbed by chromophore in the cell that causes changes in cellular level such as cell proliferation. 4 15

### Pulp Perforation and Laser Irradiation

Experimental animals were anesthetized with Ketamine HCl (0.2-cc per kg body weight) prior to preparation of a class-1 cavity on the occlusal surface of the maxillary right first molar using a low-speed round diamond bur with a diameter of 1 mm to approach the pulp chamber. Pulp roof perforation was performed using KFile no. 08 and was marked by visually observed bleeding. The treatment group was divided into eight groups consisting of a control group and a treatment group. that the control group perforated the pulp roof and directly filled with GIC, and the treatment group irradiated with a laser for 40 seconds and then filled with GIC after perforation of the pulp roof. Each group was observed on different observation days, that is, days 7 and 14, and each was observed to see the expressions of VEGF-A and TGF-β1.

### Immunohistochemistry Examination of Vascular Endothelial Growth Factor-A and Transforming Growth Factor-β1

Experimental animals from each treatment group to be treated by peritoneal injection after 7 and 14 days from the treatment. After decapitation, the jawbone in the interdental area of the maxillary right first molar was taken. Histological preparations were made through the process of fixation, dehydration and infiltration, purification, paraffin infiltration, embedding, sectioning, and sticking to the object glass. The preparations are then checked to see if the tissue cuts made are right at the perforation location. The next step was immunohistochemical examination using the monoclonal antibody anti-VEGF-A antibody (11B5) ab38909 (Abcam) and anti-TGF-β1 antibody (TB21) ab27969 (Abcam). The slide was blocked with 3% H _2_ O _2_ in phosphate-buffered saline (PBS) incubation for 20 minutes and at room temperature. Slides were washed with PBS pH of 7.4 and blocked with 1% bovine serum albumin (BSA) in PBS for 60 minutes. Slide was labeled with a primary antibody anti-VEGF-A or TGF-β1 in 1% BSA overnight at 4°C. Slides were washed with PBS pH of 7.4 thrice for 5 minutes. The slide labeled with secondary antibody goat antirat immunoglobulin (Ig)-G biotin for 1 hour at room temperature. The washing was done thrice for 5 minutes with PBS pH of 7.4. Slide incubation was performed with SA-HRP (streptavidin-horseradish peroxidase) 1:500 for 40 minutes at room temperature. Slides were washed with PBS pH of 7.4 thrice for 5 minutes. The slide was dripped with substrate chromogen DAB (diamino benzidine tetrahydrochloride 3.3) for 20 minutes. Slides were washed with PBS pH of 7.4 thrice for 5 minutes and proceeded with dH _2_ O thrice each for 5 minutes. Counterstain was performed with methyl green 1% at room temperature. The slide was soaked with tap water for 5 minutes and dried overnight at room temperature. Mounting and cover with a cover glass, and then observed with a light microscope at ×400 and ×1,000 magnification, counting 10 fields of view.

### Statistical Analysis

Data were tabulated and analyzed using SPSS statistical software for Windows, Version 23.0 (IBM SPSS Statistics for Windows, Version 23.0. Armonk, New York, United States: IBM). Differences in the mean value of VEGF-A and TGF-β1 were analyzed statistically by one-way analysis of variance (ANOVA) at a 95% significance.

## Results

### Effect of Laser Irradiation to Vascular Endothelial Growth Factor-A Expression at Days 7 and 14

**Fig. 2:**
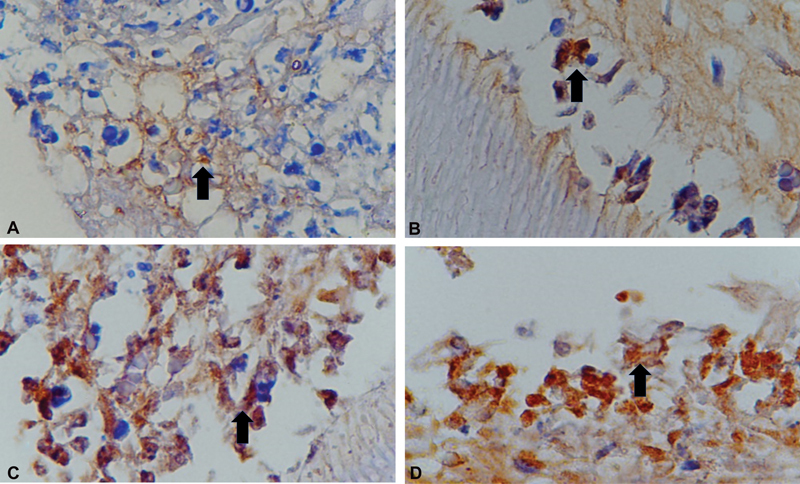
Immunohistochemical staining of VEGF-A expression (×400 magnification, Nikon H600L microscope, DS Fi2 camera 300 megapixels). ( **A** ) Fibroblast cells at control group day 7. ( **B** ) Fibroblast cells at control group day 14. ( **C** ) Fibroblast cells at laser group day 7. ( **D** ) Fibroblast cells at laser group day 14. VEGF, vascular endothelial growth factor.

Immunohistochemistry (IHC) examination is done by counting cells that expressed VEGF-A from each group. Data examination of VEGF-A expression on the dental pulp healing was done at days 7 and 14 with immunohistochemical staining showed brown cell color. The number of brown cells was calculated and compared between the control group and laser irradiation. The using of laser irradiation with Dentolaser 650 nm on dental pulp for 40 seconds showed VEGF-A expression on day 14 higher compared with day 7, and control group (days 7 and 14) by IHC examination ( Fig. 2 ).

**Fig. 3:**
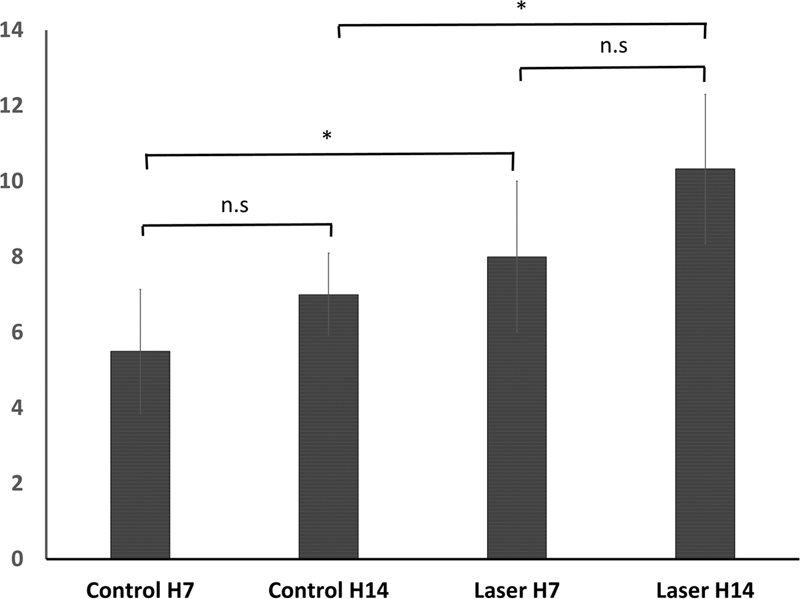
Difference of VEGF-A expression at observation day 7 and 14 in IHC examination. *Significantly different with *α* = 0.05. IHC, immunohistochemistry; n.s, not significant; VEGF, vascular endothelial growth factor.

There was elevation of VEGF-A expression from days 7 to 14 in laser and control groups ( Fig. 3 ). Table 1 showed that there was a significant difference in the VEGF-A expression within group ( *p* = 0.001), but there was no significant difference in VEGF-A expression between control group and laser in the days 7 ( *p* = 0.092) and 14 ( *p* = 0.092).

**Table 1:** Mean and standard deviation (SD) of VEGF-A expression in IHC examination

Group	VEGF-A (X ± SD) a	*p* -Value b
Control H7	5.5 ± 1.643	0.001
Control H14	7 ± 1.095	
Laser H7	8 ± 2.000	
Laser H14	10.33 ± 1.966	

Abbreviations: IHC, immunohistochemistry; SD, standard deviation; VEGF, vascular endothelial growth factor.

a = mean.

b *p* -Value: significance level of 0.05.

### Effect of Laser Irradiation to Transforming Growth Factor-β1 Expression at Days 7 and 14

**Fig. 4:**
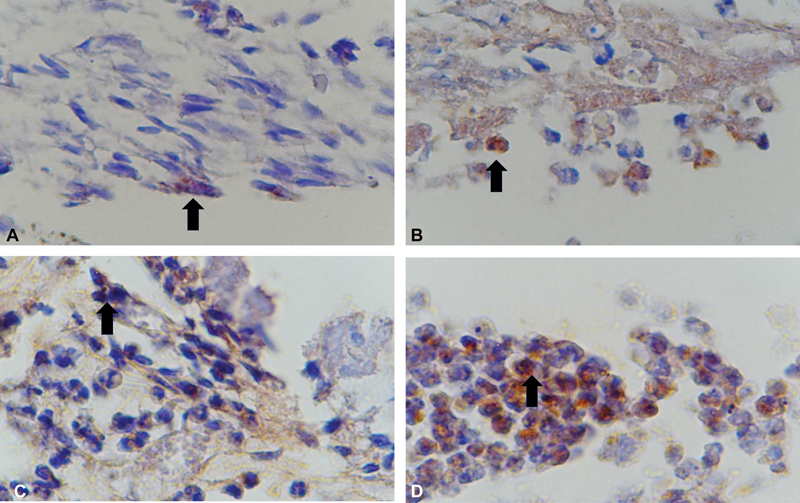
Immunohistochemical staining of TGF-β1 expression (×400 magnification, Nikon H600L microscope, DS Fi2 camera 300 megapixels). ( **A** ) Fibroblast cells at control group day 7. ( **B** ) Fibroblast cells at control group day 14. ( **C** ) Fibroblast cells at laser group day 7. ( **D** ) Fibroblast cells at laser group day 7.TGF, transforming growth factor.

**Fig. 5:**
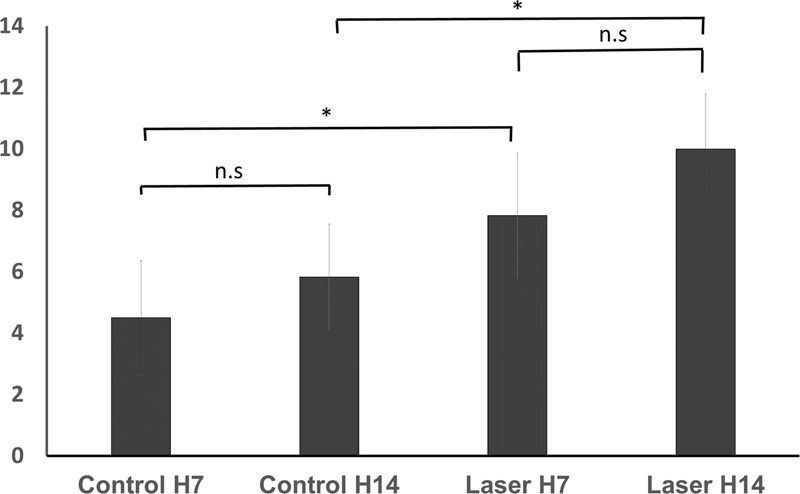
TGF-β1 expression at day 7 and 14 in IHC examination on pulp fibroblast. *Significant difference with *α* = 0.05. IHC, immunohistochemistry; n.s, not significant; TGF, transforming growth factor.

Data examination of TGF-β1 expression on the dental pulp healing was done at days 7 and 14 with immunohistochemical staining showed brown cell color. The number of brown cells was calculated and compared between the control group and laser irradiation. IHC staining examination on dental pulp after treated with Dentolaser 650 nm shows increasing TGF-β1 expression at day 14 compared with day 7 and control group (days 7 and 14; Fig. 4 ). Expression of TGF-β1 in control group showed no significant increasing between days 7 and 14 ( *p* = 0.228), even though in the laser group ( *p* = 0.79). There was a significant difference on TGF-β1 expression between control and laser groups ( *p* = 0.000; Table 2 ). TGF-β1 expression is significantly higher in laser group compared with control on day 7 ( *p* = 0.015). At day 14, significant difference also found in laser group compared with control group ( *p* = 0.02; Fig. 5 ).

**Table 2:** Mean and standard deviation (SD) of TGF-β1 expression in IHC examination

Kelompok	TGF-β1 (X ± SD) a	*p* -Value b
Control H7	4.5 ± 1.871	0.000
Control H14	5.83 ± 1.722	
Laser H7	7.83 ± 2.041	
Laser H14	10 ± 1.789	

Abbreviations: IHC, immunohistochemistry; SD: standard deviation; TGF, transforming growth factor.

a = mean.

b *p* -Value: Significance level of 0.05.

## Discussion

Present study is similar with a study conducted by Alghamdi et al that stated laser irradiation with red or near-red wavelength (500–1,200 nm) has biostimulator effect. Laser with energy density 0.05 to 10 J/cm ^2^ can induce proliferation and laser with energy density >10 J/cm ^2^ can induce antiproliferation effect. This study was conducted using laser with energy density 2.2 J/cm ^2^ , so it can induce proliferation. 9

In this study, it was found that the expression of VEGF-A and TGF-β1 increased significantly in the laser group, both on days 7 and 14. This significant difference means that the use of a 650-nm diode laser increases the expression of VEGF-A and TGF-β1. This is in accordance with the theory that LLLT can increase the modulation of tissue repair processes by stimulating cellular reactions such as migration, proliferation, apoptosis, and cell differentiation. These results are also similar with studies using a laser with a wavelength of 635 nm, where it was concluded that the use of lasers can increase cell proliferation seen from the expression of VEGF-A and TGF-β1. 16 17

LLLT, including a 650-nm diode laser, acts on the mitochondria of cells. LLLT stimulates photochemical reactions in cells, a process called biostimulation or photo-biomodulation. When photon light is absorbed by the chromophores inside the cell, the electrons in the chromophores are excited and jump from low-energy orbits to high-energy orbits. This stored energy can be used by the system for several cellular tasks. Diode lasers increase adenosine triphosphate (ATP) production, modulate reactive oxygen species (ROS), and induce transcription factors. Some of transcription factors are regulated in changes in cellular redox reactions. These will cause protein synthesis that ends with cell proliferation and migration, modulation of cytokines, growth factors, inflammatory mediators, and increased tissue oxygenation. Some of them associated with AP1, cFos and cJun heterodimers, nuclear factor kappa B (NF-kB), p53, activating transcription factors/cAMP response element binding protein (ATF/CREB), hypoxia inductible factor (HIF)-1, and HIF-like factor. 6 18

Increased ROS activates transcription factors that cause upregulation of genes that play a role in cell proliferation and migration, cytokine production, and growth factor. 6 The NF-kB pathway is activated by cytokine receptors, such as TNF-α and interleukin (IL). Cytokines secreted by T-lymphocyte cells, which play a role in the process of forming dentin bridge, are TNF as a proinflammatory cytokine and TGF as an anti-inflammatory cytokine. The balance between the two cytokines is what affects the thickness of the αβ dentin bridge formed. TGF can induce proliferation and differentiation of β-stem cell pulp into odontoblast-like cells. 19 20

The TGF-β/suppressor of mothers against decapentaplegic (SMAD) pathway is one of the pathways that influences the proliferation and differentiation of odontoblast-like cells. SMAD is a protein that is expressed in odontoblast-like cells. SMADs-2, -3, and -4 are activated after TGF-β1 signaling. TGF-β1 induces phosphorylation of SMAD2 and SMAD3. SMAD3 is required by TGF-β1 to induce FGF-2. 15 17

Under hypoxia conditions, VEGF expression increases due to the bond between HIF-1 which activates VEGF mRNA transcription. VEGF can also be induced from the influence of IL-6, IL-8, endothelins, calcium ions, nitric oxide, and TGF-β. VEGF-A has several receptors, one of which is VEGFR2. VEGF plays a role in the process of angiogenesis, leading to increase permeability of blood vessel membranes, proliferation, and migration of endothelial cells. 6 18

VEGF-A expression on control group in day 14 is higher than VEGF-A expression in day 7. In theory of wound healing process, VEGF starts showing at day 3 after injury and is upregulated until day 7. VEGF expression will decrease in day 13 and after 3 weeks is getting normal. 17 21 This is similar to the result of this study in VEGF-A group, in control and laser groups, VEGF-A expression is increasing from days 7 to 14. In treated with laser group, the escalation of VEGF-A expression in laser group is higher than the escalation in control group. This showed that laser irradiation can increase VEGF-A expression in reparative dentinogenesis process.

The increase of TGF-β1 expression in laser group is bigger than the escalation of TGF-β1 expression in control group which means TGF-β1 expression is increasing physiologically in repair process after injury happens, but laser irradiation can accelerate the repair process. Results of this study is linear to other study that using propolis and calcium hydroxide as pulp capping agent. Those studies stated that TGF-β1 expression is higher on day 14 compared with TGF-β1 expression on day 7, both in control and treatment groups. 22 TGF-β1 is a multifunction cytokine that plays important role in repair process after injury. 1 TGF-β1 acts in inflammation process, recruitment of cell progenitor, proliferation, and cell differentiation. This study is limited to 14 days of observation where TGF-β1 expression is increased, similar with graphic of wound healing process. After that, in differentiation stage, antifibrinogenic factor such as stratifin will be released, so that avoid over healing and keloid. 21

## Conclusion

Diode laser 650 nm with 40-second irradiation time shows increment from days 7 to 14 reflecting increase in pulp healing by modulating VEGF-A and TGF-β1 expression since days 7 to 14.
